# ﻿Two new species of *Trilacuna* Tong & Li, 2007 (Araneae, Oonopidae) from Yunnan Province, China, with a key to all known *Trilacuna* species of Yunnan

**DOI:** 10.3897/zookeys.1248.158273

**Published:** 2025-08-04

**Authors:** Chengzhi Dai, Qiang Chen, Yanfeng Tong, Dongju Bian, Shuqiang Li

**Affiliations:** 1 College of Life Science, Shenyang Normal University, Shenyang 110034, Liaoning, China Shenyang Normal University Shenyang China; 2 Institute of Applied Ecology, Chinese Academy of Sciences, Shenyang 110016, China Institute of Applied Ecology, Chinese Academy of Sciences Shenyang China; 3 College of Life Sciences, Anhui Normal University, Wuhu, Anhui 241000, China Anhui Normal University Anhui China

**Keywords:** Biodiversity, goblin spiders, identification key, morphology, taxonomy

## Abstract

Two new species of the genus *Trilacuna* Tong & Li, 2007, *T.manhao* Tong & Li, **sp. nov.** (♂♀) and *T.mopanshan* Tong & Li, **sp. nov.** (♂♀), are described from Yunnan, China. Descriptions, diagnoses, photomicroscopy images and a key to species of Yunnan Province are provided.

## ﻿Introduction

The spider family Oonopidae, commonly referred to as goblin spiders, represents one of the most diverse families, currently comprising 1964 extant species in 115 genera (WSC 2025). The genus *Trilacuna* Tong & Li, 2007, currently comprises 51 valid species, with a broad geographic range extending from Iran to the Korean Peninsula and south to Sumatra, Indonesia (WSC 2025). Currently, 30 species of this genus are known to occur in China, of which 13 from Yunnan, seven are from Chongqing, four are from Xizang, two are from Guizhou, one is from Anhui and Zhejiang, and three are from Fujian, Hebei and Sichuan Provinces, respectively (Ma et al. 2024; Tong et al. 2024; Shi et al. 2025).

**Figure 1.**
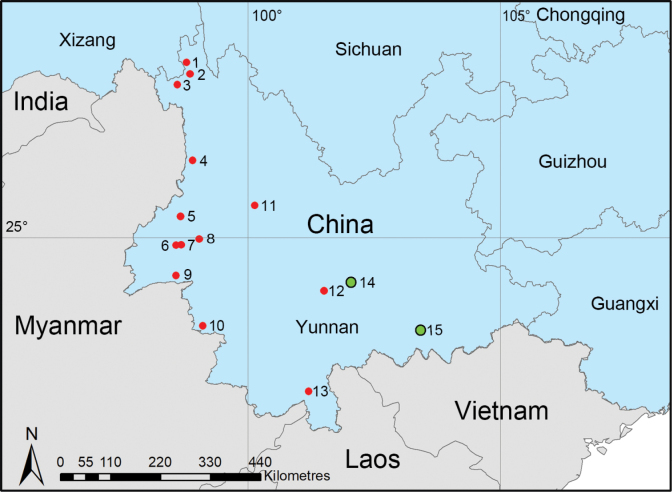
Distribution records of *Trilacuna* species from Yunnan Province, China: red circles represent 13 known species, green circles indicate the two new species. 1. *T.rastrum*; 2. *T.cuneata*; 3. *T.gongshan*; 4. *T.fugong*; 5. *T.datang*; 6. *T.wuhe*; 7. *T.longling*; 8. *T.bawan*; 9. *T.xiaoheishan*; 10. *T.wumanshan*; 11. *T.cangshan*; 12. *T.xinping*; 13. *T.aoxian*; 14. *T.mopanshan* sp. nov.; 15. *T.manhao* sp. nov.

Yunnan, located in the far southwest of China, is a province renowned for its breathtaking natural landscapes and rich biodiversity. The highest species diversity of the genus *Trilacuna* is concentrated in Yunnan Province. To date, 13 species have been recorded in Yunnan, primarily distributed in the western regions (Tong and Li 2007; Liu et al. 2019; Tong et al. 2019; Ma et al. 2023; Tong et al. 2024; see Fig. 1).

In this paper, two new species of *Trilacuna* from central and southeastern Yunnan Province are described. Detailed morphological descriptions, diagnostic photographs, and an identification key to the species of Yunnan are provided.

## ﻿Material and methods

The specimens were examined under a Leica M205 C stereomicroscope. Fine details were studied under an Olympus BX51 compound microscope. Endogynes were cleared in lactic acid. Photomicroscope images were taken with a Canon EOS 750D zoom digital camera (24.2 megapixels) mounted on the Olympus BX51. Raw photos were first stacked with Helicon Focus v. 8.2.0 to get the composite images, which were then processed in Adobe Photoshop CC 2020. Scanning electron microscope images (SEM) were taken under high vacuum with a Hitachi S-4800 after critical-point drying and gold-palladium coating. The distribution map was generated with ArcGIS v. 10.2 (ESRI Inc.). All measurements were taken using the Olympus BX51 and are in millimeters. Taxonomic descriptions follow Tong et al. (2020). Type material is deposited in the Shenyang Normal University (**SYNU**) in Shenyang, Liaoning Province, China (curator: Yanfeng Tong).

The following abbreviations are used in the text and figures: **ALE** = anterior lateral eyes; **ap** = apodemes; **as** = anterior sclerite; **bb** = basal branch; **bll** = blade-like lobes; **blp** = basal leaf-shaped projection; **glo** = globular structure; **lb** = lateral branch; **lll** = leaf-like lobes; **mb** = median branch; **PLE** = posterior lateral eyes; **psp** = posterior spiracle; **sar** = sclerotized, recurved arches; **tba** = transverse bars; **tsc** = transverse sclerite.

## ﻿Taxonomy

### ﻿Family Oonopidae Simon, 1890

#### ﻿Genus Trilacuna

Taxon classificationAnimaliaAraneaeOonopidae

Tong & Li, 2007

F134A6AF-9D43-57A3-885F-09D1E571444A

##### Type species.

*Trilacunarastrum* Tong & Li, 2007 from Yunnan, China.

##### Diagnosis.

See Tong et al. (2020).

##### Composition.

Fifty-three species, including two described here.

##### Distribution.

From Iran to the Korean Peninsula and south to Sumatra, Indonesia.

### ﻿Key to species of the genus *Trilacuna* occurring in Yunnan, China

Females of *T.cangshan*, *T.cuneata* and *T.wumanshan* are unknown.

**Table:** 

1 (0)	Males	**2**
—	Females	**16**
2 (1)	Epigastric region elevated (Fig. 7E; Liu et al. 2019: fig. 1E; Tong et al. 2019: figs 1I, 4I, 7I, 19I; Ma et al. 2023: fig. 5C; Tong et al. 2024: fig. 5H)	**3**
—	Epigastric region flat	**11**
3 (2)	Carapace with deep depressions on posterior surface; epigastric region with 2 rows of short, black thorn-like setae and cluster of short setae (Tong et al. 2024: fig. 5A, B, E)	***T.aoxian* Tong & Li, 2024**
—	Without above-mentioned characters	**4**
4 (3)	Epigastric region with long wedge-shaped scape (Liu et al. 2019: fig. 1E, F)	***T.cuneata* Tong, 2019**
—	Epigastric region without long wedge-shaped scape	**5**
5 (4)	Epigastric region with 4 long, very thick setae; sternum with many short, slanting thick setae (Tong et al. 2019: fig. 4E, I)	***T.datang* Tong, Zhang & Li, 2019**
—	Without above-mentioned characters	**6**
6 (5)	Sternum rugose (Fig. 7D)	**7**
—	Sternum smooth or reticulate	**8**
7 (6)	Palpal femur enlarged, nearly globular; bulbal base strongly swollen ventrally; psembolus with basal branch (Fig. 9A, C, H)	***T.mopanshan* sp. nov.**
—	Palpal femur elongated; bulbal base not swollen; psembolus without basal branch, but with row of combs (Ma et al. 2023: fig. 6A, D, G, J)	***T.xiaoheishan* Tong, Yang & Zhang, 2023**
8 (6)	Sternum with several grooves on posterior part (Tong et al. 2019: fig. 1E)	***T.bawan* Tong, Zhang & Li, 2019**
—	Sternum without grooves on posterior part	**9**
9 (8)	Sternum with cluster of short setae on posterior part; psembolus with comb-like prolateral lobes (Tong et al. 2019: figs 19E, 20B)	***T.xinping* Tong, Zhang & Li, 2019**
—	Without above-mentioned characters	**10**
10 (9)	Psembolus with rows of lobes in basal ventral groove and large distal plate (Tong et al. 2019: figs 7E, 8C)	***T.fugong* Tong, Zhang & Li, 2019**
—	Without above-mentioned characters, but with cluster of short fiber structures and large dorsal plate (Ma et al. 2023: fig. 4J)	***T.wumanshan* Tong, Yang & Zhang, 2023**
11 (2)	Eyes vestigial, only visible in frontal view; sternum reticulate (Ma et al. 2023: fig. 1A, D–F)	***T.cangshan* Tong, Yang, & Zhang, 2023**
—	Eyes normal; sternum smooth or rugose	**12**
12 (11)	Sternum rugose, with 2 clusters of short, thick setae on posterior part (Fig. 1D)	***T.manhao* sp. nov.**
—	Sternum smooth, without thick setae	**13**
13 (12)	Sternum with many rows of small ridges on posterior area (Tong et al. 2019: figs 13E, 16E)	**14**
—	Sternum without small ridges on posterior area	**15**
14 (13)	Psembolus with 4 long, finger-like lobes and 2 distal broad prolateral lobes (Tong et al. 2019: fig. 17B)	***T.wuhe* Tong, Zhang & Li, 2019**
—	Psembolus with 2 narrow, blade-like prolateral lobes (Tong et al. 2019: fig. 14B)	***T.longling* Tong, Zhang & Li, 2019**
15 (13)	Endites distally slightly branched; psembolus with 3 long, tooth-like prolateral lobes (Tong et al. 2019: figs 10D, 11E)	***T.gongshan* Tong, Zhang & Li, 2019**
—	Endites distally strongly branched; psembolus without 3 long, tooth-like prolateral lobes (Tong and Li 2007: figs 3, 7–10)	***T.rastrum* Tong & Li, 2007**
16 (1)	Carapace with deep depressions on posterior surface (Tong et al. 2024: fig. 6A, B)	***T.aoxian***
—	Carapace without deep depressions on posterior surface	**17**
17 (16)	Surface of sternum smooth (Fig. 10D)	**18**
—	Surface of sternum rugose (Tong et al. 2019: figs 15E, 18E)	**23**
18 (17)	Endogyne with posterior horseshoe-shaped sclerite (Tong et al. 2019: fig. 9B)	***T.fugong***
—	Endogyne without horseshoe-shaped sclerite	**19**
19 (18)	Endogyne with only transverse bar (Tong et al. 2019: fig. 25D)	***T.xinping***
—	Endogyne with 2 transverse sclerites, i.e., anterior transverse sclerite and a posterior transverse bar	**20**
20 (19)	Recurved arches of epigastric region weakly sclerotized (Tong et al. 2019: fig. 24G, H)	***T.gongshan***
—	Recurved arches of epigastric region strongly sclerotized (Tong et al. 2019: fig. 24A)	**21**
21 (20)	Transverse sclerite of endogyne straight; ALE separated by about 1.0 times their diameter; clypeus height about 1.1–1.3 times ALE diameters (Figs 6D, 10H; Tong et al. 2019: figs 3H, 24B)	**22**
—	Transverse sclerite of endogyne curved; ALE separated by about 1.3 diameters; clypeus height about 2 ALE diameters (Tong and Li 2007: fig. 5)	***T.rastrum***
22 (21)	Dorsal scutum covering about 5/6 of abdomen length (Fig. 10A)	***T.mopanshan* sp. nov.**
—	Dorsal scutum covering about whole abdomen length	***T.bawan***
23 (16)	Sternum slightly rugose on middle part (Tong et al. 2019: figs 15E, 18E)	**24**
—	Sternum strongly rugose across whole surface (Fig. 5D)	**25**
24 (23)	Middle part of anterior margin of postgastric scutum smooth (Tong et al. 2019: fig. 24I)	***T.longling***
—	Middle part of anterior margin of postgastric scutum arch-shaped (Tong et al. 2019: fig. 25A)	***T.wuhe***
25 (23)	Lateral surface of carapace granulated (Fig. 5B, F)	***T.manhao* sp. nov.**
—	Lateral surface of carapace smooth	**26**
26 (25)	ALE separated by less than radius; middle part of posterior margin of epigastric scutum curved (Ma et al. 2023: fig. 7D, G)	***T.xiaoheishan***
—	ALE separated by more than diameter; middle part of posterior margin of epigastric scutum straight (Tong et al. 2019: figs 6D, 24C)	***T.datang***

#### ﻿ Trilacuna manhao

Taxon classificationAnimaliaAraneaeOonopidae

Tong & Li sp. nov.

8A45ED60-E999-53BA-8E19-B3AC179E2B60

https://zoobank.org/21E4CC48-7520-40BB-A391-DBD3AB61E96A

**Figure 2.**
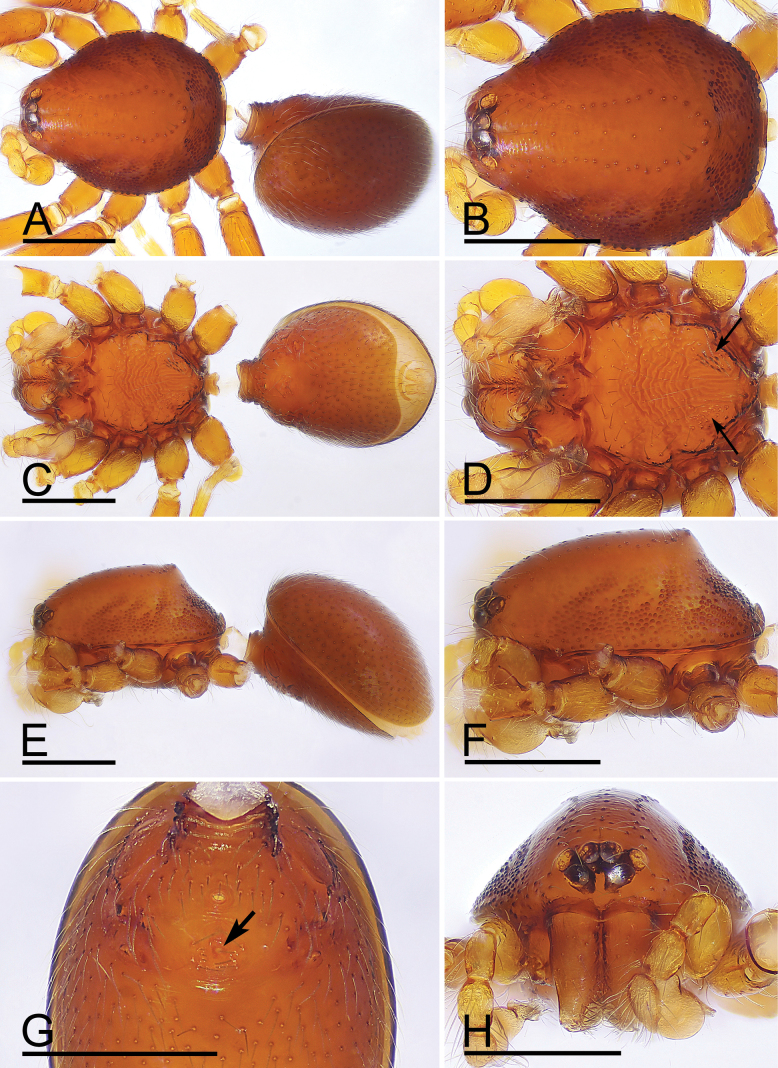
*Trilacunamanhao* sp. nov., male holotype. A, C, E. Habitus, dorsal, ventral and lateral views; B, D, F, H. Prosoma, dorsal, ventral, lateral and anterior views, arrows in Fig. 1D show the short, thick setae; G. Abdomen, ventral view, arrow shows the small spot. Scale bars: 0.4 mm.

**Figure 3.**
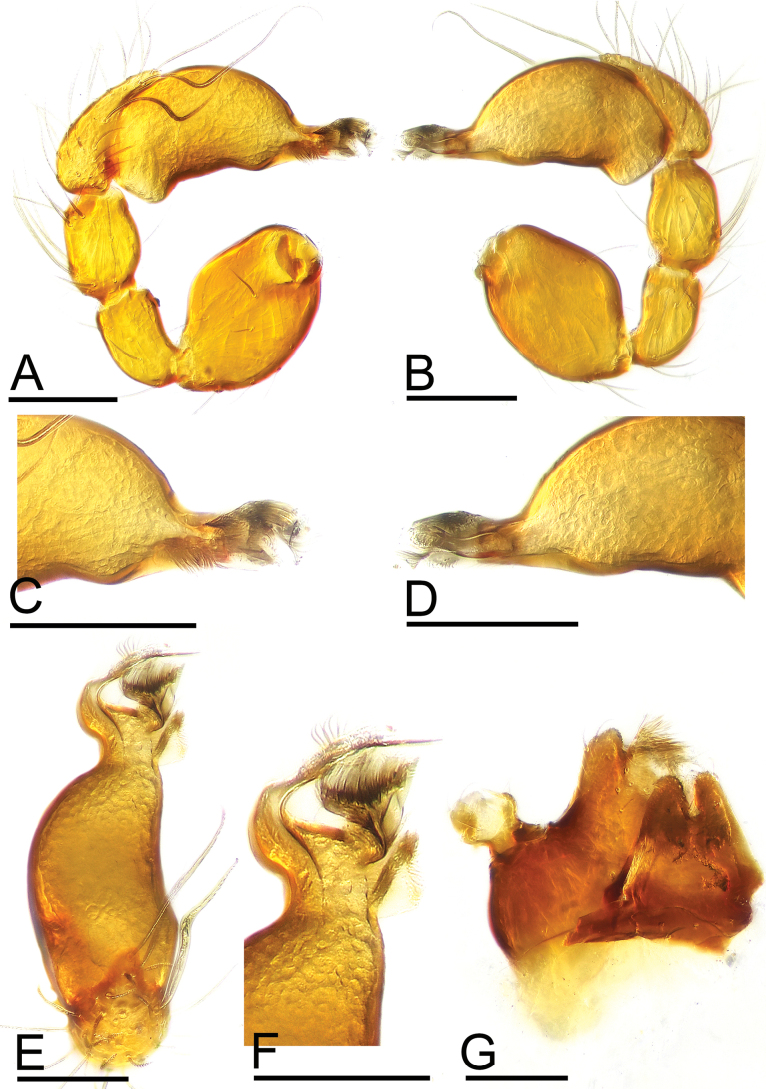
*Trilacunamanhao* sp. nov. A–F. Male left palp; A, B, E. Prolateral, retrolateral and dorsal views; C, D, F. Distal part of bulb, prolateral, retrolateral and dorsal views; G. Labium and endite, ventral view. Scale bars: 0.1 mm.

**Figure 4.**
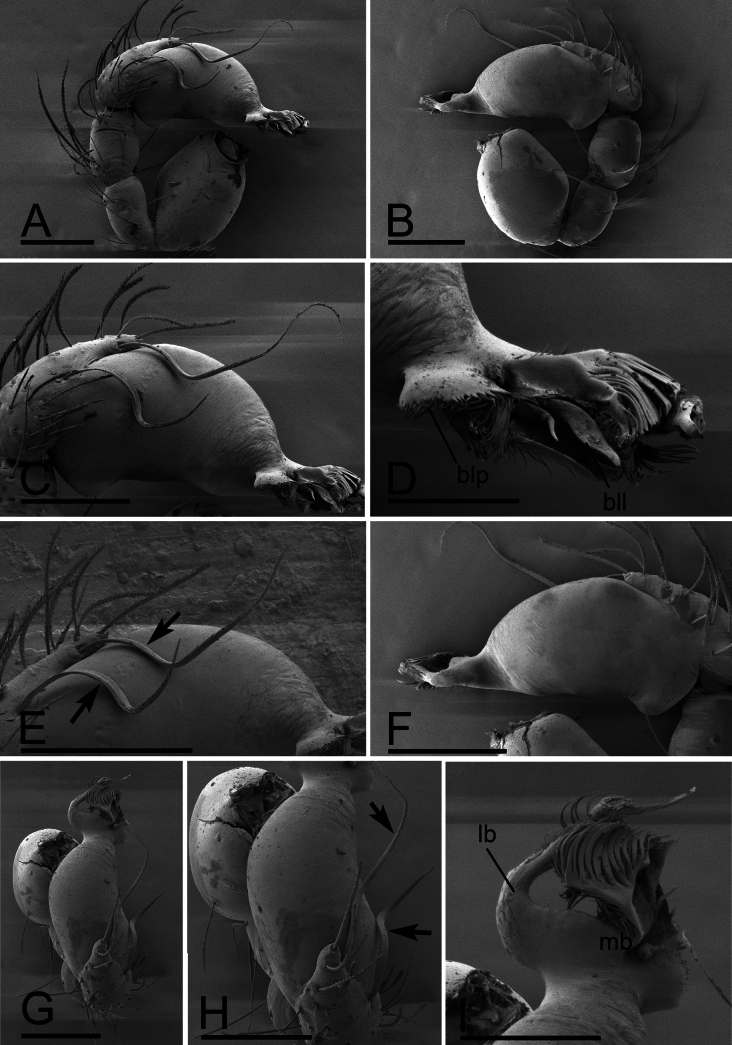
*Trilacunamanhao* sp. nov., male left palp. A, B, G. Prolateral, retrolateral and dorsal views; C, F. Bulb, prolateral and retrolateral views; D, I. Distal part of bulb, prolateral and dorsal views; E, H. Detail of cymbium, arrows show the flat setae. Abbreviations: bll = blade-like lobes; blp = basal leaf-shaped projection; lb = lateral branch; mb = median branch. Scale bars: 0.1 mm (A–C, E–H); 0.05 mm (D, I).

**Figure 5.**
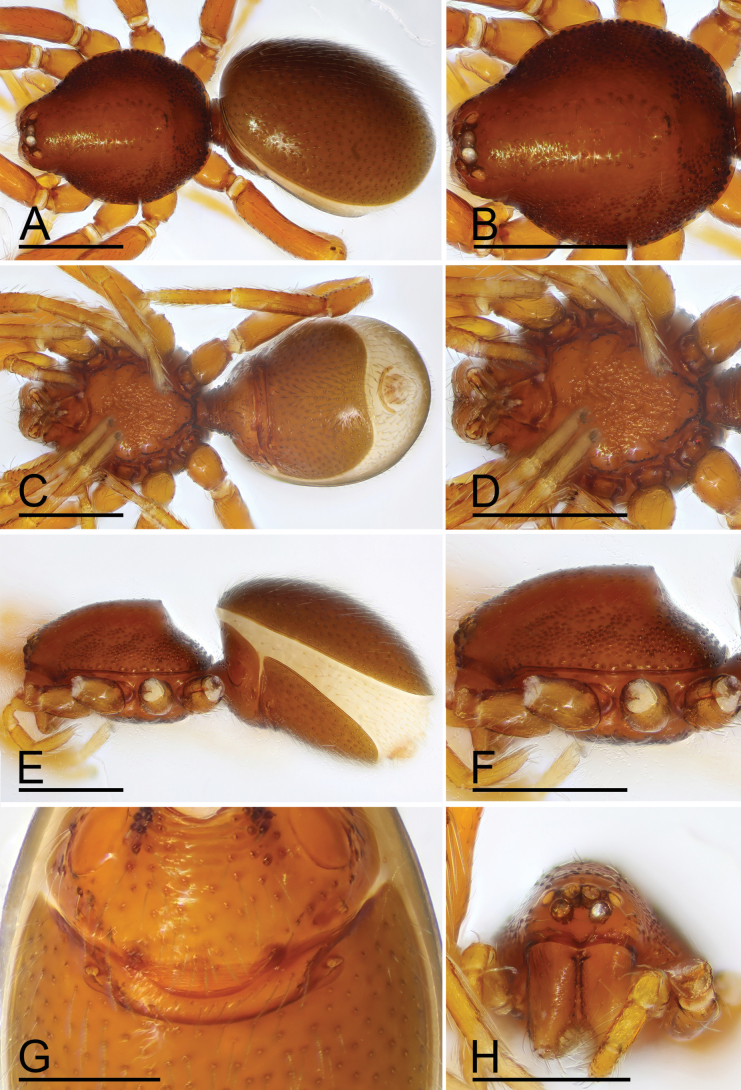
*Trilacunamanhao* sp. nov., female paratype. A, C, E. Habitus, dorsal, ventral and lateral views; B, D, F, H. Prosoma, dorsal, ventral, lateral and anterior views; G. Epigastric area, ventral view. Scale bars: 0.4 mm.

**Figure 6.**
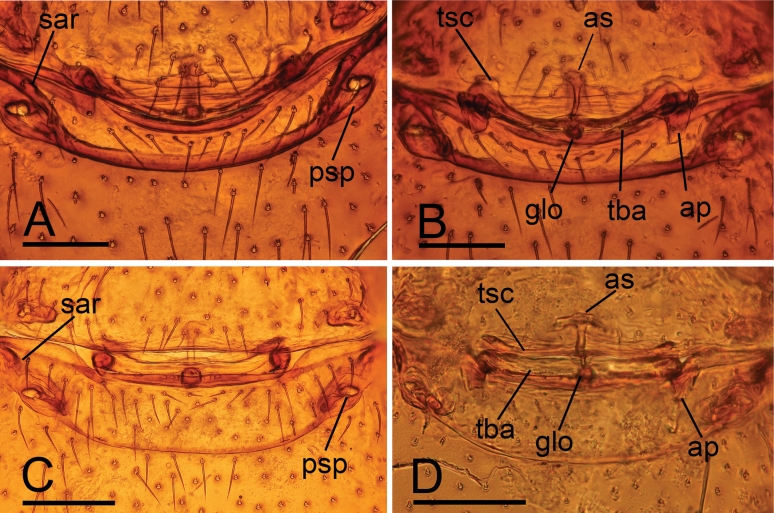
*Trilacuna* spp., endogyne. A, B. *Trilacunamanhao* sp. nov.; C, D. *Trilacunamopanshan* sp. nov.; A, C. Ventral view; B, D. Dorsal view. Abbreviations: ap = apodeme; as = anterior sclerite; glo = globular structure; psp = posterior spiracle; sar = sclerotized, recurved arches; tba = transverse bars; tsc = transverse sclerite. Scale bars: 0.1 mm.

Figs 2 , 3 , 4 , 5 , 6A, B Common name: 蔓耗三窝蛛

##### Type material.

***Holotype*** China • ♂ (SYNU-902); Yunnan Prov., Honghe Hani and Yi Auton. Pref., Gejiu City, Manhao Town, Lvshuihe Tropical Rainforest Reserve; 23°1'32.1"N, 103°24'3.78"E, 477 m elev.; 19.V.2015; Z. Chen & Y. Li leg. ***Paratypes*.** China • 3♀ (SYNU-F-3618–3620); Yunnan Prov., Gejiu City, Manhao Town, Lvshuihe Tropical Rainforest Resort; 23°2'32.64"N, 103°24'44.28"E, 498 m elev.; 2.VI.2018; X. Zhang leg.

##### Etymology.

The specific name is a noun in apposition taken from the type locality.

##### Diagnosis.

The new species is similar to *T.longling* in the shape of bulb, the small dark spot on male epigastric region and the rugose sternal surface, but can be distinguished by the sternum with two clusters of short, thick setae on posterior part (Fig. 2D) vs. lacking, but with many rows of small ridges (Tong et al. 2019: fig. 13E), the cymbium with two extremely long, flat setae (Fig. 4A, C, E, H) vs. lacking (Tong et al. 2019: fig. 14A), and the anterior margin of female postgastric scutum smoothly curved (Fig. 6A) vs. with a small notch at middle (Tong et al. 2019: fig. 24I).

##### Description.

**Male.** Body: yellow-brown, legs yellow; habitus as in Fig. 2A, C, E; body length 1.48. Carapace (Fig. 2B, F): 0.74 long, 0.59 wide; sides granulate. Eyes (Fig. 2B, H): well developed; ALE largest, PLE smallest; posterior eye row recurved from above, procurved from front; ALE separated from edge of carapace by 1.1 times diameters. Mouthparts (Figs 2D, 3G): endites distally branched. Sternum (Fig. 2D): surface strongly rugose, with two clusters of short, thick setae. Abdomen (Fig. 2A, C, E, G): 0.69 long, 0.54 wide; sperm pore situated in front of anterior spiracles; apodemes present, posterior spiracles connected by shallow groove; with small spot situated between anterior and posterior spiracles. Palp (Figs 3A–F, 4A–I): yellow; 0.49 long (0.17, 0.09, 0.09, 0.14); femur swollen (width/length = 0.71); tibia about as long as patella; cymbium with 2 very long, curved, flat setae; bulb kidney-shape, base slightly swollen ventrally, tapering apically; psembolus complex, with basal leaf-shaped projection (blp), 1 blade-like lobe (bll), broad median branch (mb) and lateral branch (lb), surrounded by numerous fiber structures.

**Female.** Same as male except as noted. Body length 1.56; habitus as in Fig. 5A, C, E. Carapace 0.74 long, 0.59 wide. Abdomen: 0.85 long, 0.67 wide. Epigastric area (Figs 5G, 6A): with recurved, strongly sclerotized arches (sar). Endogyne (Fig. 6B): with narrow, curved transverse sclerite (tsc); with anterior slender stick-shaped sclerite (as) and posterior small globular structure (glo); transverse bars (tba) with pair of short lateral apodemes (ap).

##### Distribution.

Known only from the type locality, Yunnan Province, China (Fig. 1).

#### ﻿ Trilacuna mopanshan

Taxon classificationAnimaliaAraneaeOonopidae

Tong & Li sp. nov.

82ED2FAD-FD29-5B1F-96B4-46F67F3B01DF

https://zoobank.org/6AADB417-59E5-461B-AFD3-E3BDA5CA143B

**Figure 7.**
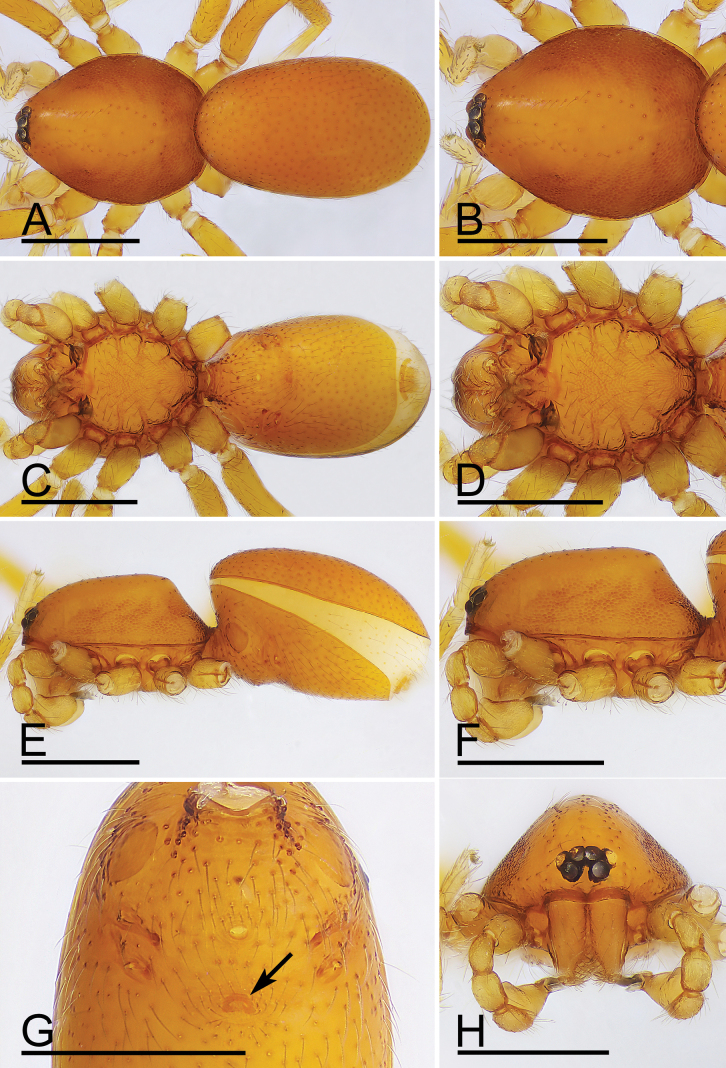
*Trilacunamopanshan* sp. nov., male holotype. A, C, E. Habitus, dorsal, ventral and lateral views; B, D, F, H. Prosoma, dorsal, ventral, lateral and anterior views; G. Epigastric area, ventral view, arrow shows the semi-circular spot. Scale bars: 0.4 mm.

**Figure 8.**
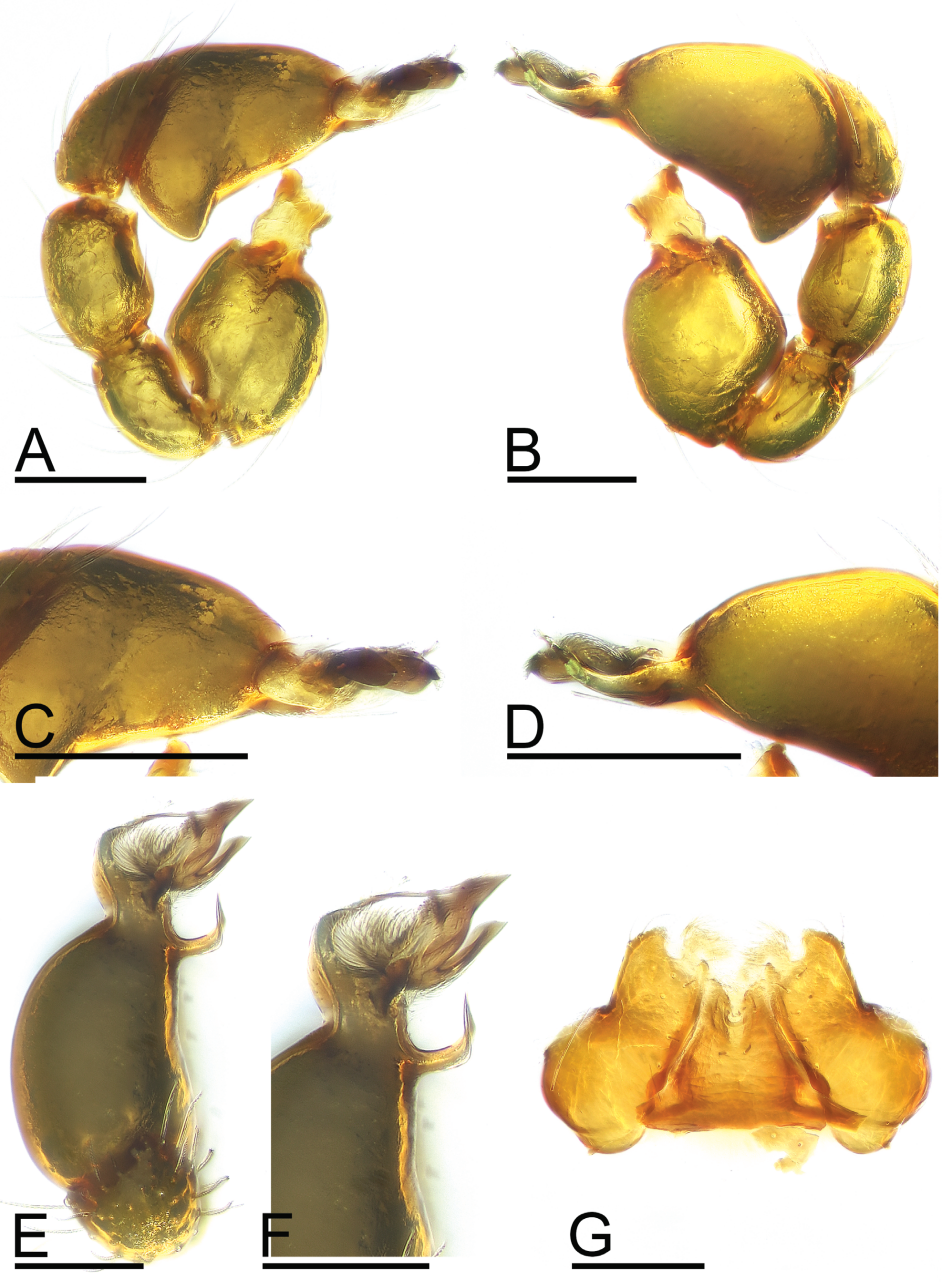
*Trilacunamopanshan* sp. nov. A–F. Male left palp; A, B, E. Prolateral, retrolateral and dorsal views; C, D, F. Bulb, prolateral, retrolateral and dorsal views; G. Labium and endite, ventral view. Scale bars: 0.1 mm.

**Figure 9.**
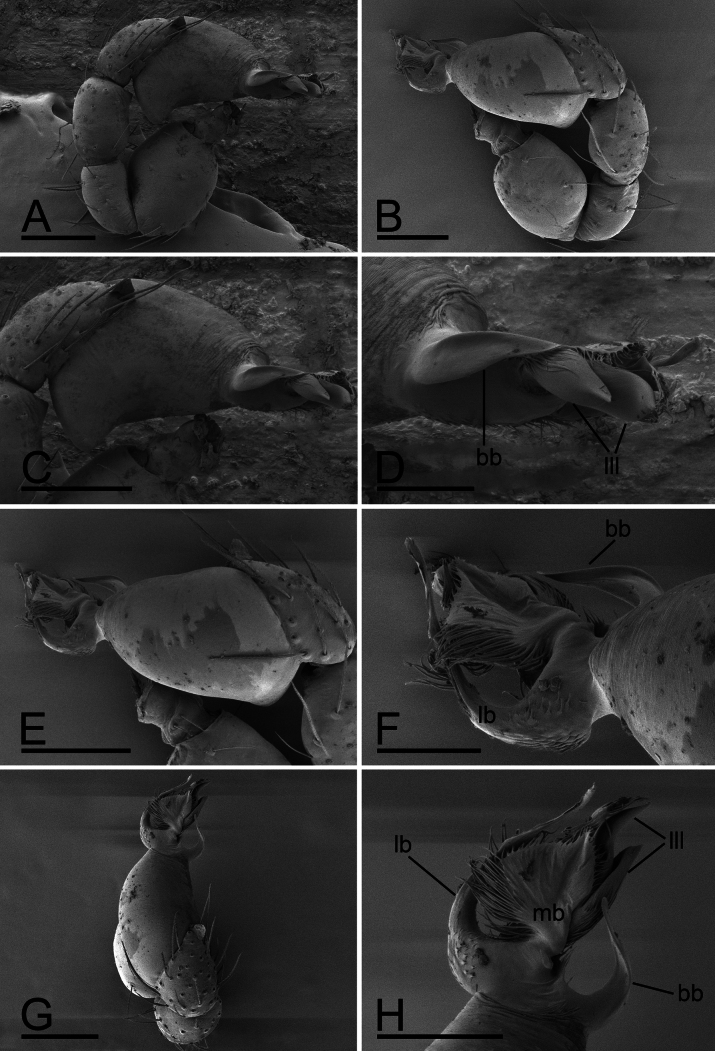
*Trilacunamopanshan* sp. nov., male left palp. A, B, G. Prolateral, retrolateral and dorsal views; C, E. Bulb, prolateral and retrolateralviews; D, F, H. Distal part of bulb, prolateral, retrolateral and dorsal views. Abbreviations: bb = basal branch; lb = lateral branch; lll = leaf-like lobes; mb = median branch. Scale bars: 0.1 mm (A–C, E, G); 0.05 mm (D, F, H).

**Figure 10.**
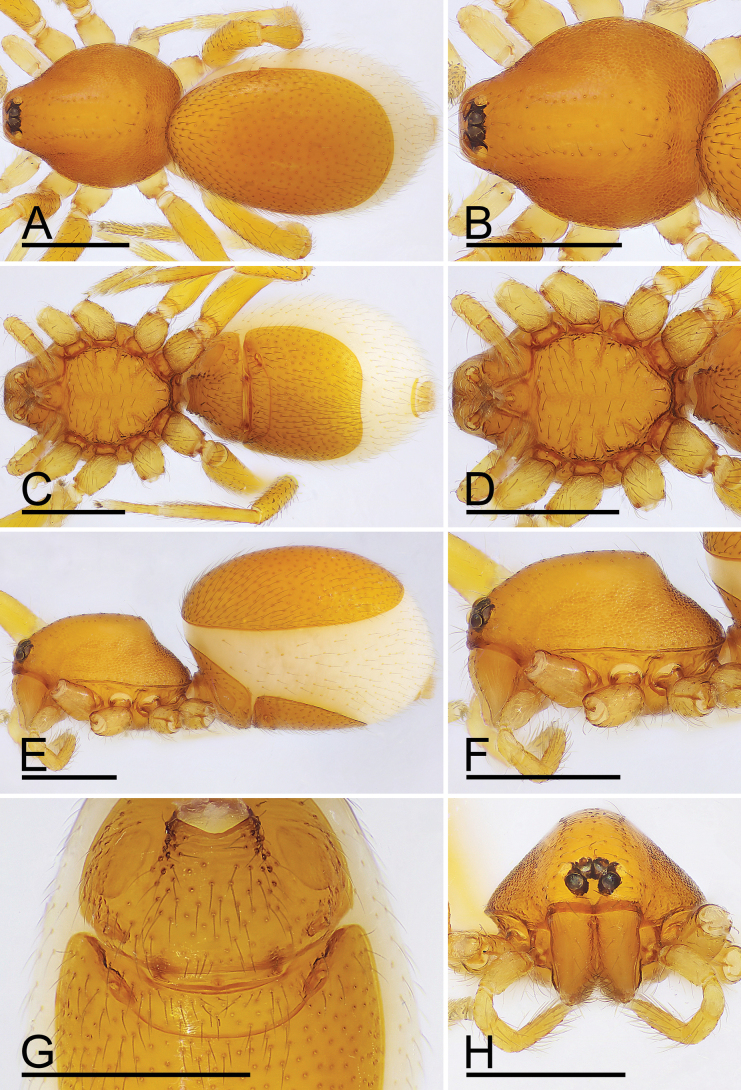
*Trilacunamopanshan* sp. nov., female paratype. A, C, E. Habitus, dorsal, ventral and lateral views; B, D, F, H. Prosoma, dorsal, ventral, lateral and anterior views; G. Epigastric area, ventral view. Scale bars: 0.4 mm (A–F, H); 0.2 mm (G).

Figs 6C, D , 7 , 8 , 9 , 10 Common name: 磨盘山三窝蛛

##### Type material.

***Holotype*** China • ♂ (SYNU-925); Yunnan Prov., Yuxi City, Xinping Co., Mopanshan National Forest Park; 23°57'36.42"N, 101°56'23.58"E, 2113 m elev.; 1.VI.2015; Z. Chen & Y. Li leg. ***Paratypes*.** China • 6♀ (SYNU-926, SYNU-980–984); same data as holotype.

##### Other material.

China • 1♂3♀ (SYNU-985–988); Yunnan Prov., Yuxi City, Xinping Co., Xinhua Township, Guzhou Wild Forest; 24°6'37.98"N, 101°50'59.88"E, 1987 m elev.; 2.VI.2015; Z. Chen & Y. Li leg.

##### Etymology.

The specific name is a noun in apposition taken from the type locality.

##### Diagnosis.

The new species is similar to *T.xinping* in the granulated carapace and the slightly elevated male epigastric region, but can be distinguished by the sternum strongly rugose (Fig. 7D) vs. reticulate, with a cluster of short setae (Tong et al. 2019: fig. 20A), the base of bulb strongly swollen ventrally (Figs 8A, 9A) vs. straight (Tong et al. 2019: figs 19E, 23E), the psembolus with two leaf-like lobes (Fig. 9D) vs. comb-shaped lobes (Tong et al. 2019: fig. 20B), and the scutum of female covering about 5/6 of abdomen length (Fig. 10A, C, E) vs. the whole abdomen (Tong et al. 2019: fig. 21A, B, C).

##### Description.

**Male.** Body: yellow, legs pale yellow; habitus as in Fig. 7A, C, E; body length 1.52; Carapace (Fig. 7B, F): 0.75 long, 0.52 wide; sides granulate. lateral sides granulate. Eyes (Fig. 7B, H): well developed; ALE largest, PLE smallest; posterior eye row recurved from above, procurved from front; ALE separated from edge of carapace by 1.3 diameters. Mouthparts (Figs 7D, 8G): endites distally branched. Sternum (Fig. 7D): surface strongly rugose. Abdomen (Fig. 7A, C, E, G): 0.69 long, 0.54 wide; sperm pore situated at level of anterior spiracles; apodemes present, posterior spiracles not connected by groove; with small semi-circular spot situated between posterior spiracles; epigastric region slightly elevated. Palp (Figs 8A–F, 9A–H): yellow; 0.54 long (0.17, 0.10, 0.11, 0.16); femur swollen (width/length = 0.82); tibia about as long as patella; bulb triangular, base strongly swollen ventrally, tapering apically; psembolus complex, with basal branch (bb), 2 leaf-like lobes (lll), broad median branch (mb) and lateral branch (lb), surrounded by numerous fiber structures.

**Female.** Same as male except as noted. Body length 1.62; habitus as in Fig. 10A, C, E. Carapace 0.68 long, 0.55 wide. Abdomen: 0.85 long, 0.62 wide; dorsal scutum covering about 5/6 of abdomen length. Epigastric area (Figs 10G, 6C): with recurved, strongly sclerotized arches (sar). Endogyne (Fig. 6D): with narrow, straight transverse sclerite (tsc); with anterior T-shaped slender sclerite (as) and posterior small globular structure (glo); transverse bars (tba) with pair of short lateral apodemes (ap).

##### Distribution.

Known only from the type locality, Yunnan Province, China (Fig. 1).

## Supplementary Material

XML Treatment for Trilacuna

XML Treatment for Trilacuna manhao

XML Treatment for Trilacuna mopanshan

## ﻿Additional information

### Use of AI

No use of AI was reported.

### Author ORCIDs

Chengzhi Dai https://orcid.org/0009-0009-2416-4943

Qiang Chen https://orcid.org/0009-0007-9722-1561

Yanfeng Tong https://orcid.org/0000-0002-4348-7029

Dongju Bian https://orcid.org/0000-0002-9324-8026

Shuqiang Li https://orcid.org/0000-0002-3290-5416

### Data availability

All of the data that support the findings of this study are available in the main text.

## References

[B1] Liu S Yu S Tong Y (2019) A new species of the genus *Trilacuna* (Araneae, Oonopidae) from Yunnan Province, China. Acta Arachnologica Sinica 28(1): 47–51. 10.3969/j.issn.1005-9628.2019.01.005

[B2] Ma J Bian D Tong Y Yang Z Zhang Z (2023) Three new species of the genus *Trilacuna* Tong & Li, 2007 (Araneae, Oonopidae) from Yunnan Province, China. ZooKeys 1174: 289–300. 10.3897/zookeys.1174.106130 37614247 PMC10442693

[B3] Ma J Chen Q Tong Y (2024) Four new species of the genus *Trilacuna* Tong & Li, 2007 (Araneae, Oonopidae) from China. ZooKeys 1219: 331–346. 10.3897/zookeys.1219.138793 39677512 PMC11645522

[B4] Shi S Bian D Tong Y Li S (2025) First record of the genus *Trilacuna* Tong & Li, 2007 (Araneae, Oonopidae) from Xizang, China, with descriptions of three new species and one newly recorded species. ZooKeys 1229: 213–232. 10.3897/zookeys.1229.145844 40061156 PMC11886598

[B5] Tong Y Li S (2007) One new genus and four new species of oonopid spiders from south west China (Araneae: Oonopidae). Annales Zoologici, Warszawa 57: 331–340.

[B6] Tong Y Chen H Bai S Zhang Z Li S (2019) Seven new species of the genus *Trilacuna* Tong & Li, 2007 from Yunnan, China (Araneae, Oonopidae). ZooKeys 821: 11–44. 10.3897/zookeys.821.29599 PMC6367306 30740019

[B7] Tong Y Li S Bian D (2020) Taxonomic studies on the genus *Trilacuna* (Araneae, Oonopidae) from Myanmar. ZooKeys 960: 39–62. 10.3897/zookeys.960.54053 32884397 PMC7445191

[B8] Tong Y Shao Y Bian D Li S (2024) Two new oonopid spiders (Arachnida, Araneae) from Xishuangbanna tropical rainforest, Yunnan, China. ZooKeys 1205: 333–348. 10.3897/zookeys.1205.124183 38984213 PMC11231571

[B9] WSC (2025) World Spider Catalog. Version 26. Natural History Museum Bern. http://wsc.nmbe.ch [Accessed on 7 May 2025]

